# Clinical service use as people with Attention Deficit Hyperactivity Disorder transition into adolescence and adulthood: a prospective longitudinal study

**DOI:** 10.1186/s12913-016-1509-0

**Published:** 2016-07-11

**Authors:** Hanna Eklund, Tim Cadman, James Findon, Hannah Hayward, Deirdre Howley, Jennifer Beecham, Kiriakos Xenitidis, Declan Murphy, Philip Asherson, Karen Glaser

**Affiliations:** Department of Forensic and Neurodevelopmental Sciences, Institute of Psychiatry, Psychology & Neuroscience, King’s College London, De Crespigny Park, London, SE5 8AF UK; Personal Social Services Research Unit, Cowdray House, London School of Economics and Political Sciences, Houghton Street, London, WC2A 2AE UK; Behavioural Genetics Unit, Institute of Psychiatry, Psychology & Neuroscience, King’s College London, London, SE5 8AF UK; MRC Social Genetic and Developmental Psychiatry, Institute of Psychiatry, Psychology & Neuroscience, King’s College London, De Crespigny Park, London, SE5 8AF UK; Institute of Gerontology, Department of Social Sciences, Health and Medicine, King’s College London, Strand, London, WC2R 2LS UK

**Keywords:** Clinical service use, Attention deficit hyperactivity disorder, Transition, Adolescence, Young adulthood

## Abstract

**Background:**

While Attention Deficit Hyperactivity Disorder (ADHD) often persists into adulthood, little is known about the needs and service use among adolescents and young adults with ADHD. The present study followed-up a cohort diagnosed with ADHD as children and assessed their: 1) needs, 2) correlates of contact with clinical services, and 3) experiences of transition from child to adult health services.

**Methods:**

Ninety one young people aged 14–24 were recruited from the UK subset of the International Multi-Centre ADHD Genetics (IMAGE) Project. Affected young people and parents conducted face-to-face interviews and self-completion questionnaires including a modified version of the Client Services Receipt Inventory, The Barkley’s ADHD rating scale, The Clinical Interview Schedule-Revised, and the Zarit Burden Interview. Changes in key need characteristics (e.g. ADHD symptoms and impairments) over a 3-year period were examined using fixed effect models. Generalised Estimating Equations (GEE) were used to explore how key characteristics (such as ADHD symptoms) were associated with contact with clinical services across the three years.

**Results:**

At baseline 62 % met diagnostic criteria for ADHD and presented with a range of ADHD related impairments, psychiatric comorbidities, and significant caregiver burden. While ADHD symptoms and related impairments lessened significantly over the three years, psychiatric comorbidities and caregiver burden remained stable. The strongest correlate of contact with clinical services was age (OR 0.65 95 % CI 0.49–0.84) with the odds of reported contact with clinical services decreasing by 35 % for each year increase in age at baseline and by 25 % for each year increase in age over time. Only 9 % of the sample had experienced a transfer to adult services, with the majority reporting unmet needs in healthcare transition.

**Conclusions:**

Despite continuing needs, few were in contact with adult health services or had received sufficient help with transition between child and adult health services. The main determinant of health service use for adolescents and young adults with ADHD is age – not needs. Service models should address the needs of ADHD individuals who are no longer children.

**Electronic supplementary material:**

The online version of this article (doi:10.1186/s12913-016-1509-0) contains supplementary material, which is available to authorized users.

## Background

Attention Deficit Hyperactivity Disorder (ADHD) is a common neurodevelopmental disorder (affecting 6–7 % of the population) that often persists into adulthood. Up to two-thirds of ADHD children still warrant the diagnosis in adulthood [1] and this significantly impacts on life [2, 3]. For instance, adults with ADHD are more likely than the general population to: 1) be unemployed [4] and experience more frequent job losses [4]; 2) under achieve in education [5]; 3) experience greater instability in emotional relationships [6]; 4) display anti-social and offending behaviours [7] and 5) suffer from drug abuse, anxiety and depression [8].

However, despite continuing symptoms and needs, few young adults with ADHD or their families are in receipt of treatments [9–12]. In fact, adolescents and young adults with ADHD are among those least likely to transfer from child to adult mental health services [13]. This is not due to a lack of clear recommendations for the management of ADHD in adulthood that now exist in several guidelines [14–17]. A number of interventions are available to support children and families affected by ADHD. For example, National Institute for Clinical Excellence (NICE) guidelines recommend parent training/education programs and psychological interventions such as CBT as the first-line treatment, with medication to be used in severe cases or where these treatments have proven ineffective [14]. In childhood, this support is normally provided by Child and Adolescent Mental Health Services (CAMHS).

Likely reasons for the low use of adult health services among those with ADHD include a lack of (or difficulty accessing) appropriate services during this transitional period alongside individual and caregiver factors that are unique to this developmental stage [18–22]. For example, adolescents’ increasing treatment drop-out and medication non-adherence rates may be attributable to both declining parental control over their health behaviour, and negative attitudes toward mental health treatment [18–21, 23, 24]. However, to date little research has been carried out into this issue (and, to our knowledge, no studies so far have investigated correlates of health services use among young people with a childhood diagnosis of ADHD now at the transition into adolescence and young adulthood).

The research that does exist has been carried out mostly in children with ADHD and has identified multiple factors that are associated with service use. These include high levels of caregiver burden, low levels of caregiver network support, comorbid psychiatric symptoms, functional impairments of the child and parent or teacher rated ADHD symptom severity [25–33]. A few studies have also reported on the association between the lower educational level of the mother and health services among children with ADHD [28, 29]; however, to date, there is scarce evidence relating to health service use among adolescents and young adults with ADHD. To our knowledge, only one previous study has examined correlates of health service use among adolescents with ADHD. This study reported on the follow-up of children with a high risk of ADHD status in the United States and found that increased parental network support, ADHD symptoms, caregiver burden and lower stigma perceptions among youth were independently associated with increased health service use [23]. However, as this study was limited to a small part of Florida it is not known whether these findings generalise to ADHD populations in Europe. Furthermore, as the above study used screening tools rather than diagnostic assessments to determine ADHD status, the findings may vary significantly when stricter diagnostic criteria are applied.

Nevertheless, there is prior evidence to suggest that factors other than clinical severity (such as ADHD symptom severity) may be more important for health service use among adolescents and young adults with ADHD. In one study, using the UK General Practice Research Database to examine prescriptions of drugs to ADHD patients aged 15–21 years, drug prescribing fell steeply at the age of 17 with an almost complete disengagement from services by the age of 21 [10]. It seems unlikely that such a sudden fall in drug prescribing is the result of an abrupt symptom remission, suggesting instead an important role played by age in influencing drug treatment. A better picture of what determines health service use among adolescents and young adults is therefore warranted and is vital not only to better design and implement appropriate and effective care programmes, but to support family members who are likely to be integral to the care of this patient group [34].

However, in order to devise appropriate interventions for adolescents and young adults with ADHD it is not only necessary to consider the correlates of health service use but also to identify the needs and unmet needs of young people with ADHD during this transitional period. Healthcare transition refers to the process that starts with preparing a patient to leave a health-care setting and finishes when the patient is received in the next setting [35]. NICE guidelines recommend that a person with ADHD receiving treatment and care from CAMHS or paediatric services should be reassessed at school leaving age to establish the need for continuing treatment into adulthood. If they continue to have significant symptoms of ADHD or other coexisting conditions that require treatment then arrangements should be made for a smooth transition to adult services with details of the anticipated treatment and services that they young person will require. While NICE states that the precise timing of arrangements may vary locally they recommend that this should be planned in advance and should usually be completed by the time the person is 18 years [14].

Although transition planning and management is recognised as a key element in the organisation and delivery of health services [36–38], for many young people with mental health problems, transition to adult health services is poorly planned, lacks co-ordination and results in discontinuity of care [39]. Given that most mental illnesses in adult life begin in adolescence, it has been reported that the current child-adult split in mental health services creates a systematic weakness when need is most pressing [39, 40], Furthermore, the situation for young people with ADHD has been identified as especially difficult [41]. For example, one study suggested that only about 15 % of young people with ADHD in England make the transition to adult services [42], further highlighting the challenges parents are likely to encounter in trying to support their children [34].

To date, very few studies have investigated healthcare transition among young people with ADHD. A recent scoping review of transition from child to adult mental health services for young people with ADHD identified only four studies which have sought to evaluate the journey of young people with ADHD between mental health services, despite a clear need for transition being identified [24]. More longitudinal studies and service evaluations are therefore warranted to truly understand and identify what is needed and currently available for successful transition to appropriate adult services for ADHD patients and to provide an overall picture to successful integration to adult services [24, 43, 44].

Therefore, for the first time, we explored the experiences of the healthcare transition (i.e. the process of moving from child to adult health services) from both the young person’s and parent’s perspectives and investigated both needs and contact with clinical services among those with a childhood diagnosis of ADHD who were now at transition to adolescence and young adulthood.

## Methods

### Design, setting and participants

This prospective longitudinal study was conducted at the young person’s family home in England or at the Institute of Psychiatry in London, with all travel expenses reimbursed. Around half of young people and their families lived in Greater London while the remainder were spread throughout England from Cornwall to Lincolnshire in the North East. All young people aged 14 to 24 in the IMAGE research database were invited by the research team to participate in the study. Young people and parents were each sent separate letters of invitation and information sheets explaining the study together with an update form to state their interest in the study together with a stamped addressed return envelope. Invitation letters and information sheets explained (i) the rationale of the study; (ii) what the study would involve (i.e., a face-to-face interview lasting about 2 h including the completion of a self-completion questionnaire, and a joint interview with both the parent and the young person assessing ADHD symptoms, needs, health service use and healthcare transition), and (iii) the confidential aspects of the study (i.e., that the young person and parent would remain blind to each other’s responses given during their individual interviews with the interviews being carried out in two separate rooms; that both the young person and the parent would be identified by a number and not by name; that the data gathered would remain confidential solely to be used for research purposes, and would not be shared with anyone apart from those directly involved in the research study).

We included 91 families consisting of young people aged 14 to 24 (*n* = 91) and one of their parents (mostly mothers, *n* = 91). Families were recruited through their child’s childhood clinical diagnosis of combined type ADHD from the UK subset of the International Multi-Centre ADHD Genetics (IMAGE) Project research database. Families had originally been recruited to the IMAGE study by referral from child and adolescent clinics in the southeast of England on the basis that they had received a clinical diagnosis of combined type ADHD (as defined in the DSM-IV) and had at least one surviving biological sibling aged 5–17 years of age. ADHD probands and their siblings were included in the IMAGE study if they were between the ages of 5–17, had an IQ of 70 or higher, were of European or Caucasian descent, and had at least one biological parent willing to provide DNA samples. As the IMAGE study was a molecular and genetic study participants were excluded if they had been diagnosed with: autism, epilepsy, IQ below 70, brain disorders, and/or any genetic or medical disorder associated with externalising behaviours that might mimic ADHD based on both history and clinical assessment.

Families were followed-up over a 3 year period with data collection beginning in March 2009 and ending in January 2013, with an average follow-up time of 2.35 years (SD = 0.89). Significant efforts were placed on participant retention, for instance repeated attempts to contact families. Thus only 18 % (17/91) of our sample contributed only one year’s observation (*n* = 15) or did not have young person-parent interviews for at least two waves (*n* = 2). Diagnostic threshold for ADHD was defined using the informant version of the Barkley’s ADHD rating scale for adults [45] with scores of 6 or greater being defined as exceeding the DSM-IV threshold and thus being clinically significant. We also measured ADHD symptoms at the start of the study using the DIVA [46], a diagnostic interview measure for adults with ADHD recommended by the European ADHD Consensus group [15]. A comparison of the DIVA and Barkley’s scale showed no significant difference in those who met the diagnostic threshold for ADHD at baseline (72 % versus 62 % respectively, *χ*^2^ = 3.35, *p* = 0.07).

### Measures

Face-to-face structured interviews and self-completion questionnaires were administered to both the young person and their parent at each time point. We used the Andersen-Newman behavioural model of service use to investigate correlates of clinical service use [47, 48]. The Andersen-Newman model is an established framework widely used by health economists, psychologists, and medical sociologists to explain patterns of service utilisation among diverse populations [49, 50]. The model conceptualises service use as a function of predisposing (such as the person’s age), enabling (such as parental education) and need factors (such as the frequency and severity of ADHD related symptoms and impairments, psychiatric comorbidities, and caregiver burden).

We used a modified version of the Client Services Receipt Inventory (CSRI) to capture service use and transitions from child to adult services [51]. This version was adapted by Beecham and co-authors in 2009 to young people with ADHD and included a series of questions on service use and transitions. We asked the young person and his/her parent whether the young person was being seen by child or adult health services, with those answering yes categorised as being in contact with clinical services (coded as 1). As relatively few were in contact with services it was necessary to combine those in touch with any type of services into one category. Other questions included whether the service was a child, adult or non-age specific service, if they had moved (or were currently planning a move) from a child to an adult health service, and if they had received (or were currently receiving) any support from health services in regards to moving to adult health services. The nature of, and need for, support from services was also captured including whether young people and parents were satisfied with the support received during the move from child to adult services. Detailed questions regarding help received or needed were also asked of both young people and parents with responses recorded in terms of help not needed yet, got enough help, and needed or need more help. Finally, young people and parents were asked to give an overall rating of services they had received to indicate if they were very satisfied, satisfied, not satisfied, or not at all satisfied.

We also used the modified version of the CSRI to capture predisposing and enabling factors. Measures included the socio-economic and demographic backgrounds of both the young person and parent. We considered age to be a predisposing characteristic and parent’s education, an enabling factor. Parent’s education was categorised into two groups: those with low educational levels (i.e. A levels/higher education certificate or below) versus those with high educational levels (i.e. higher education diploma or above).

We used several different measures of need including indicators of the severity and frequency of ADHD symptoms and related impairments, psychiatric comorbidities, and parent caregiver burden. We used the informant version of the Barkley’s ADHD rating scale for adults administered to the parent to measure current (i.e. in the previous six months) ADHD symptoms (as described above) and related impairments [45]. This scale measures ADHD related impairments across 10 different areas of functioning including home life, occupation, social interactions, education, driving, money management, leisure time and routine daily responsibilities. The parent was asked to rate the frequency and severity of their child’s current impairments with scores on a 4 point scale ranging from 0 to 3 capturing the severity and frequency of the behaviours representing ‘not at all or rarely’,’sometimes’, ‘often’ and ‘very often’. For those parents who responded ‘often’ and ‘very often’ their children were categorised as having a difficulty in that area in contrast to all others who were categorised as not having a difficulty. As some young people were or had been on medication for ADHD symptoms in the previous six months, we asked the parent to report ADHD symptoms as if their child was off medication to minimise under or over reporting of symptoms.

Psychiatric co-morbidities were assessed using The Clinical Interview Schedule-Revised (CIS-R) [52], a standardised, valid, and reliable structured diagnostic instrument used for rating psychiatric symptoms in both clinical and general adult populations both clinical and general adult populations [53, 54]. This was administered to the young person to assess symptoms across 14 domains (e.g., anxiety, depression). Symptoms are regarded as severe when the score is 2 or more on each section with a total score of 12 or more being regarded as a clinically significant indicator of general mental health problems [52]. This instrument was used as to our knowledge there is no single measure of psychiatric comorbidities for use in both adolescent and adult populations.

Caregiver burden was measured using the Zarit Burden Interview—12 Item [55]. It captures the psychological and social impact of caring, and asked parents to rate the extent to which they agreed or disagreed with statements regarding their feelings about the results of caring for their child on a five-point scale, with possible responses ranging from 0 (“never”) to 4 (“nearly always”). A total score (0–48) was calculated by summing the scores for each question [55]. This short-form version of the scale has been shown to have excellent reliability and validity [56].

### Analyses

Descriptive statistics at baseline were produced to explore the demographic characteristics of the sample, need characteristics, experiences of healthcare transition and contact with clinical services. Logistic regression was conducted to investigate the predisposing, enabling and need factors associated with contact with clinical services (that is, whether or not the young person was in contact with child, adult or non-age specific services at baseline). In this and all subsequent models (described below) the score reflecting the frequency and severity of ADHD symptoms was used rather than the binary measure capturing whether participants scored above or below the threshold for ADHD.

Longitudinal analyses were then conducted on the 74 families with young person-parent interviews for at least 2 time points: with young person-parent interviews at either all three waves (*n* = 53), or at least two waves (*n* = 21). Analyses (not shown) found no significant differences in any of the key characteristics between the 17 young people who were not included in the analysis for the reasons previously stated and the 74 who were. Fixed effects models were used to examine changes in key need characteristics (e.g. ADHD symptoms and impairments) over the 3-year period. We also used Generalised Estimating Equations (GEE) to explore how key characteristics (such as ADHD symptoms) were associated with contact with clinical services across the three years [57]. In doing this, the young person’s age at the start of the study (whether at wave 1 or 2) was used and the same information as described above on symptoms, impairments, psychiatric co-morbidities and caregiver burden in each of the three years. As service use is a binary outcome, a binomial distribution with a logit link was the appropriate combination (we also chose an exchange correlation structure). The types of longitudinal analyses described above are based on the number of observations rather than the number of subjects. Thus, there are 201 observations all together as 53 people contributed 159 observations (each with 3 waves of data) and 21 people contributed 42 observations (each with 2 waves of data).

The following procedure was used to account for missing data. Where less than 10 % of responses were missing for a given measure, the value was imputed from the mode of the answered questions. If more than 10 % of answers were missing from any outcome measure all data for that carer-young person pair was excluded from the analysis.

All analyses were conducted using SAS (version 9.2).

## Results

### Sample characteristics AT baseline

The average age of young people was 17.7 years and most were male (90 %), still living at home (85 %), and in education (66 %) (Table 1). Thirty eight percent of parents reported having achieved an educational level higher than A-levels. The significantly larger proportion of males in this study is broadly in keeping with previous studies which found that ADHD is more common in males than in females, with ratios ranging from 2:1 to 9:1 depending on the subset of ADHD and the setting [58–60].

**Table 1:** Descriptive Characteristics of Study Sample and Variables (*n* = 89)

Variable				Missing (n)
	%	Mean (SD)	Range	n
YP (Young person) Age		17.65 (2.41)	14–24	0
YP Male	90			0
YP White or White British	100			0
YP Education				4
Full-time	58			
Part-time	8			
Not in education	34			
PR (Parent) Education				5
Low	62			
High	38			
YP Accommodation				4
Family home	85			
Other	15			
YP ADHD symptoms (parent report)		10.74 (4.69)	0–18	2
YP ADHD symptoms above cut-off DSM-IV diagnostic threshold (parent report)	62			2
YP ADHD impairments (parent report)		4.56 (2.73)	0–10	3
YP any ADHD impairments (parent report)	91			3
YP CISR		7.75 (6.42)	0–29	5
YP CISR above cut-off (>12)	25			5
PR Zarit Caregiver Burden		17.58 (9.39)	0–38	5
YP In contact with services	55			3
Children’s service	31			
Adult service	9			
ADHD service all ages	15			

### ADHD symptoms and impairments

The majority (62 %) of young people at baseline met DSM-IV criteria for ADHD (Table 1). Ninety one percent of the baseline sample reported ADHD related impairments in at least one activity (Table 1). Overall, around two-thirds of parents reported significant impairments in their child’s management of daily responsibilities, home life, educational activities and money management; half reported impairments among their children related to work (data not shown).

### Associated psychiatric symptoms

Twenty five percent of young people at baseline scored above cut-off (score >12) on the CIS-R, indicating the likelihood of comorbid psychopathology. For example, close to a third of young people reported significant levels of worry and over one in four reported significant levels of depression (data not shown).

### Contact with clinical services

Despite a high prevalence of ADHD and associated mental health symptoms, 45 % of young people were no longer in contact with clinical services at baseline. 31 % were still in contact with children’s health services and close to 1 in 6 (15 %) reported attending an ADHD service without age boundaries (Table 1).

### Experience of healthcare transition at baseline

Nine percent (*n* = 8) of young people reported having experienced a transfer to adult care or were currently receiving help from services in preparing for this move. Only one family out of the total sample at baseline reported having received a written transition plan as recommended in clinical guidelines. Moreover, approximately 75 % of young people and parents reported that a professional had not spoken to them about the move from child to adult services, and the 25 % who responded that a professional had spoken to them about this reported that it had been only a brief conversation. Around half of all young people (and two-thirds of all parents) reported a need for more support from services in: 1) accessing information regarding which services are available when they grow up; 2) the co-ordination of transition planning, and 3) and having someone to talk to about their practical as well as emotional needs. Analyses showed no age-related differences in young person’s and parents’ experiences of transition or support received from health services in regards to moving to adult health services.

### Correlates of contact with clinical services

Table 2 shows the association between contact with clinical services and predisposing, enabling and need factors at baseline. There was a significant relationship between age and being in contact with services with those who were in touch with clinical services being significantly younger than those who were not (Table 2). In addition, among the other factors considered, only the young person’s impairments as reported by parents were significantly associated with contact with clinical services. Meeting threshold for ADHD showed no significant relationship with contact with clinical services (Table 2).

**Table 2:** Contact with clinical services by predisposing, enabling and need factors at baseline (*n* = 89)

Variable	In contact with clinical services		Sign. Test
	Yes	No	
Predisposing	Mean (S.D.) or n (%) as appropriate		
YP age	16.96 (2.17)	18.55 (2.49)	df = 84, t = 3.17 (*p* = 0.002)
Enabling			
PR - Education			
Low	28 (55)	23 (45)	df = 1, *χ* ^2^ = 0.02 (*p* = 0.88)
High	17 (57)	13 (43)	
Need			
YP met diagnostic threshold for ADHD (parent report)	32 (38)	21 (40)	df = 1, *χ* ^2^ = 1.83 (*p* = 0.0.18)
YP not meet diagnostic threshold for ADHD (parent report)	14 (45)	17 (55)	
YP ADHD Impairments (parent report)	5.07 (2.74)	3.84 (2.64)	df = 81, t = -2.06 (*p* = 0.04)
YP Psychiatric comorbidity (CISR)	8.70 (6.73)	6.54 (5.86)	df = 82, t = -1.55 (*p* = 0.13)
PR Caregiver burden	19.34 (10.39)	16.19 (7.88)	df = 79, t = -1.51 (*p* = 0.09)

Table 3 shows the results of the logistic regression model used to analyse correlates of contact with clinical services at baseline. The key outcome measure was whether or not the participant reported being currently seen by child, adult or non-age specific clinical services. The predictors captured predisposing (young person’s age), enabling (parental education) and need factors (ADHD symptoms, impairments, psychiatric comorbidities and parental caregiver burden). Age was the strongest correlate of contact with clinical services (OR = 0.65, 95 % CI 0.494–0.841, *p* = 0.01) with a one year increase in the young person’s age reducing the odds of being in contact with services by 35 %. Only one other factor, parent reported impairment was significantly associated with contact with clinical services (OR = 1.37, 95 % CI 1.042–1.806, *p* = 0.024). The proportion of variance explained by the models was 0.25 using Cox and Snell R, and 0.33 using Nagelkerke R square, indicating that between 25 and 33 % of the variance is explained by this model.

**Table 3:** Multivariate binary logistic regression model for contact with clinical service at baseline (*n* = 76)

Predictors	Unadjusted OR	95 % Confidence Intervals		Adjusted OR	95 % Confidence Intervals	
YP Age	0.75**	0.61	0.91	0.64**	0.49	0.84
PR education	0.93	0.37	2.31	0.49	0.15	1.58
YP ADHD Symptoms (parent report)	1.09	0.99	1.20	0.89	0.76	1.05
YP ADHD Impairment (parent report)	1.19*	1.00	1.40	1.37*	1.04	1.81
YP Psychiatric comorbidity (CISR)	1.06	0.98	1.14	1.05	0.96	1.15
YP Caregiver burden	1.04	0.99	1.09	1.03	0.97	1.11

Note: Full model, log likelihood ratio = 21.35, Cox & Snell R^2^ = 0.245; Nagelkerke R^2^ = 0.329

*p < 0.05, ** *p* < 0.01

### Changes over time in contact with clinical services

Table 4 shows the results of the fixed effects model which examined changes in key need characteristics over time. There was a significant decline in ADHD symptoms and impairments as well as contact with clinical services over the period considered. However, the results show no significant differences in comorbid psychiatric symptoms or caregiver burden over time (Additional file 1).

**Table 4:** Changes in key need characteristics over time fixed effects model (n = 201)

	Survey Waves			
Variables	Baseline	Wave 2	Wave 3	F test (p)
YP ADHD symptoms (parent report)	11.07	9.56	8.51	10.05 (*p* < .000)
YP ADHD Impairments (parent report)	4.49	4.00	3.44	4.54 (*p* = 0.013)
YP Psychiatric comorbidity (CISR)	8.56	8.11	7.27	1.23 (*p* = 0.295)
PR Caregiver burden	17.79	14.98	15.95	3.27 (*p* = 0.042)
YP Service use	0.59	0.36	0.43	6.91 (*p* = 0.001)

Table 5 presents the results of the GEE model. Age at the start of the study showed a significant relationship with contact with clinical services over the course of the three years. That is, those who were older at the start of the study were significantly less likely to use services across the three years. There was no significant association with year (additional analyses, not shown, revealed few differences in the use of clinical services across the three years with close to half either consistently reporting no service use or using services at all time points, and only 18 % experiencing a transition into adult services). Neither the young person’s ADHD symptoms, level of impairment nor co-morbid symptoms showed a significant relationship with clinical service use over time.

**Table 5:** Odds ratios from GEE models for contact with clinical services (*n* = 180)

Predictors	Unadjusted ORs	95 % Confidence Intervals for ORs		Adjusted ORs	95 % Confidence Intervals for ORs	
YP age at start	0.74**	0.63	0.87	0.75**	0.62	0.90
Year 1	1.90*	1.06	3.38	1.73	0.90	3.37
Year 2	0.74	0.44	1.22	0.62	0.32	1.18
PR Education start	0.89	0.42	1.86	1.22	0.54	2.76
YP ADHD symptoms	1.05	0.99	1.12	0.98	0.89	1.08
YP ADHD Impairment	1.07	0.98	1.17	1.06	0.92	1.23
YP Psychiatric comorbidity (parent report)	0.99	0.96	1.03	1.00	0.96	1.04
PR Caregiver burden	1.02	0.99	1.04	1.01	0.99	1.03

Note: Reference categories are year 3 and higher education for parents

*p < 0.05, ** *p* < 0.01

## Discussion

To our knowledge, this is the first longitudinal analysis of needs and services use among adolescents and young adults with a childhood diagnosis of ADHD. A key strength of our study includes the high retention rate (that is, 81 % of our sample had both young person-parent interviews for at least two study waves) and the use of face-to-face interviews using reliable diagnostic and outcome measures. Moreover, our use of separate face-to-face interviews with young people and their parents enabled us to gain a more comprehensive picture of the wide range of needs among this group. Another strength lies in our consideration of multiple potential factors that may be associated with contact with clinical services. This allowed us to better quantify the relative, and independent, contribution of various factors that influence service use among this clinical group. Last, our exploration of young people’s (and their parents’) experiences of healthcare transition, enabled us to identify unmet needs in healthcare delivery from the perspectives of both the affected young person and their carers, e.g. insufficient access to information, lack of co-ordination of transition, and insufficient attention to the individual needs of young people and parents.

Our finding that close to two-thirds of adolescents still warranted DSM-IV diagnostic criteria at the start of the study is comparable to a previous findings on threshold and subthreshold cases of ADHD [1]. Similarly, previous studies have established that there are developmental changes in the presentation of ADHD as seen during the course of the present study [2]. ADHD symptoms and related impairments declined during adolescence and young adulthood; however, in a significant number of cases these symptoms persisted and are likely to cause great problems to young adults [61]. For example, earlier studies have shown that when hyperactive-impulsive symptoms do persist into adulthood they can be particularly impairing, for example being seen in adult ADHD patients with addiction [62] or forensic problems. It is therefore crucial that clinicians gain a full understanding of the needs of young people during the transition from child to adult ADHD. Moreover, as our study shows there are also significant psychiatric comorbidities and caregiver burden during this transitional period which do not appear to decline, emphasising the challenges parents are likely to encounter in trying to support their children between childhood and adulthood [34]. There is high familiality of ADHD (with approximately 20 % of parents of children with ADHD having ADHD themselves and who are thus likely to also require support) [33] and therefore both child and adult clinical service providers should be mindful of the impact of possible parental ADHD on the transition process and provide clear structured support to families in transition [12].

The results of this study suggest that irrespective of the severity of ADHD symptoms, impairments, comorbidities and carer burden, contact with health services among adolescents and young adults with ADHD is mainly determined by age. The only other factor that was associated with service use was impairments. This may be caused, at least in part, to a lessening of parental control over treatments during the transition from adolescence to young adulthood which may lead to a further elevation of functional impairment or ADHD symptoms. Such declines in impairments may result in further difficulties in managing time and appointments which in turn are likely to increase the risk of disengagement with services [24]. It is important that services across all age groups are based on the needs of individuals and are dependent on goals and methods of treatment that are both meaningful and motivating for the individual [63]. This requires not only a full understanding of the needs of young people but that young people are offered choice in treatment. For example, prior studies have indicated that adolescents and young adults may prefer to communicate with their health professionals via email or text rather than speak over the phone or attend face-to-face appointments [64], and clinicians working with young people with ADHD may wish to identify those most likely to benefit from interventions from home through the use of screening measures [64].

We found that only 9 % had transferred to adult services (or received transition planning) which is broadly in keeping with a recent audit of transitional care for adolescents with ADHD in a North West England district which found that only about 15 % of young people made the transition [42]. In addition, our study found that half of all young people (and two-thirds of all parents) reported a need for more support from services in: 1) accessing information regarding which services are available when they grow up; 2) the co-ordination of transition planning; and 3) and having someone to talk to about their practical as well as emotional needs [13]. We suggest that one way to improve healthcare transitions for this group is to ensure transition protocols contain specific guidance for implementation at ground level so that they can be effectively translated into practice. These may include psycho-educational material for both young people and parents to ensure that they have access to comprehensive, impartial and appropriately written information regarding ADHD and the transition process. To improve transition planning and co-ordination, these should be developed jointly with child and adult teams and service users to ensure that sufficient attention is given to the individual concerns of young people and parents and that joint working arrangements between child and adult services are put into place [65]. Finally, we recommend that clinicians in child and adolescent services and adult services develop a feedback mechanism to better enable clinicians in child and adolescent services to be informed of the results of the transition, and clinicians in adult services to monitor how well recently transferred patients follow transition plans.

However, it is also important to acknowledge that one of the main reasons for the lack of a transition may be the lack of appropriate adult services. There are limited ADHD-specific services for adults [66], therefore the only source of support may be adult mental health services. However, given the fiscal constraints on the NHS, young people are only likely to become eligible for transition into adult Community Mental Health Teams (CMHTs) if they are suffering from significantly impairing co-morbid mental health problems [67]. Thus in addition to the above recommendations of additional information and planning, what may also be required is a redesign and investment in services for young adults with ADHD. Promising options include increasing the age-threshold of child services so that young people could continue to be supported through these key transitional years, or creating specific ADHD services for all ages [39].

Our study does have some limitations. Given that individuals in our sample had been in contact with child and adolescent mental health services in childhood, and were also participants in research studies of ADHD, our findings may not generalise to the wider population. However, given such previous experience we would expect the individuals in our sample to be more knowledgeable and motivated to access services and thus suggest that the situation in regards to transition may be even worse in the general population. Prior contact with such child and adolescent mental health services may also have resulted in a bias toward presenting with higher levels of psychological problems at follow-up in comparison to those with no previous contact with mental health services. Nevertheless, rates of psychiatric comorbidity in our study are consistent with those reported in previous studies of ADHD [68–70].

Furthermore, as only those with a childhood IQ over 70 were included it is possible that our findings represent an under-estimate of needs among those with intellectual disabilities. This is important as previous studies have shown that adults with borderline and mild intellectual disability (ID) may have more impairments and more severe ADHD symptoms in adulthood when compared to those with ADHD but no ID [71].

In addition, as this study was initially selected for genetic studies and is thus an all-White one it is not possible to say how representative the current findings are for women and/or ethnic minorities. As previous studies have indicated, there are significant variations in service use by gender and ethnicity among young people with ADHD [72–74], which may also lead to differing health beliefs and attitudes towards help-seeking. Therefore future studies may benefit from examining the role of gender, ethnicity, learning difficulties and health beliefs on needs and services use among this group.

## Conclusions

Despite continuing needs, few ADHD adolescents and young adults are in contact with health services. Currently, the main determinant of health service use among this group is age – not needs. Service models should address the needs of ADHD individuals who are no longer children and improve the planning, co-ordination and continuity of healthcare during the transition to adult services to reduce the long-term health, social and economic consequences of untreated ADHD [14].

## Abbreviations

ADHD, attention deficit hyperactivity disorder; CIS-R, clinical interview schedule-revised; CSRI, client services receipt inventory; DSM-IV, Diagnostic and Statistical Manual of Mental Disorders- Fourth Edition; GEE, generalised estimating equations; ID, intellectual disability; IMAGE, International Multi-Centre ADHD Genetics Project; SAS, statistical analysis system

## Additional files

Additional file 1: ADHD merged datasets (Combined data files for year 1, 2 and 3 of ADHD longitudinal study). (SAV 297 kb)
